# Fluorescence Resonance Energy Transfer Imaging Reveals that Chemokine-Binding Modulates Heterodimers of CXCR4 and CCR5 Receptors

**DOI:** 10.1371/journal.pone.0003424

**Published:** 2008-10-16

**Authors:** Nilgun Isik, Dale Hereld, Tian Jin

**Affiliations:** Chemotaxis Signal Section, Laboratory of Immunogenetics, National Institute of Allergy and Infectious Diseases, National Institutes of Health, Twinbrook II Facility, Rockville, Maryland, United States of America; Yale University School of Medicine, United States of America

## Abstract

**Background:**

Dimerization has emerged as an important feature of chemokine G-protein-coupled receptors. CXCR4 and CCR5 regulate leukocyte chemotaxis and also serve as a co-receptor for HIV entry. Both receptors are recruited to the immunological synapse during T-cell activation. However, it is not clear whether they form heterodimers and whether ligand binding modulates the dimer formation.

**Methodology/Principal Findings:**

Using a sensitive Fluorescence Resonance Energy Transfer (FRET) imaging method, we investigated the formation of CCR5 and CXCR4 heterodimers on the plasma membrane of live cells. We found that CCR5 and CXCR4 exist as constitutive heterodimers and ligands of CCR5 and CXCR4 promote different conformational changes within these preexisting heterodimers. Ligands of CCR5, in contrast to a ligand of CXCR4, induced a clear increase in FRET efficiency, indicating that selective ligands promote and stabilize a distinct conformation of the heterodimers. We also found that mutations at C-terminus of CCR5 reduced its ability to form heterodimers with CXCR4. In addition, ligands induce different conformational transitions of heterodimers of CXCR4 and CCR5 or CCR5^STA^ and CCR5^Δ4^.

**Conclusions/Significance:**

Taken together, our data suggest a model in which CXCR4 and CCR5 spontaneously form heterodimers and ligand-binding to CXCR4 or CCR5 causes different conformational changes affecting heterodimerization, indicating the complexity of regulation of dimerization/function of these chemokine receptors by ligand binding.

## Introduction

Chemokine receptors are members of the superfamily of G-protein-coupled receptors (GPCRs), which posse seven transmembrane domains that are interconnected by multiple extracellular and intracellular loops, and an intracellular C-terminal tail [1]. Chemokines are a large family of small proteins that mediate recruitment of leukocytes to sites of inflammation and coordinate their trafficking throughout the human body [2], [3]. Gradients of chemokines that are detected by their receptors control cell traffic in homeostasis and inflammation in vivo [3]. Chemokines regulate leukocyte function by binding to specific chemokine receptors expressed on their surface, typically leading to the activation of receptor-associated Janus tyrosine kinases (JAKs) and the heterotrimeric G-protein GαiGβγ [4]–[7]. One basic question is how different chemokine receptors receive and transduce signals from the surface of a cell on which multiple GPCRs are expressed. Initially, GPCRs were believed to signal as simple monomers [8], [9]. However, mounting evidence now indicates that many GPCRs, including several chemokine receptors, function as dimers or higher-order oligomers [8], [9].

CXCR4 and CCR5 receptors regulate leukocyte chemotaxis in inflammation and also serve in conjunction with CD4 as co-receptors for HIV entry [1], [3]. CXCR4 normally functions as the receptor for the chemokine CXCL12/SDF-1, whereas CCR5 mediates responses to several chemokines, including CCL3/MIP1-α, CCL4/MIP1-β and CCL5/RANTES [2]. CXCR4 and CCR5 are co-expressed in several leukocyte populations including lymphocyte and monocytes [3], [10]. In addition to their roles in regulating leukocyte chemotaxis, CXCR4 and CCR5 serve as the entry co-receptors for T-tropic or M-tropic strains of HIV virus, respectively. Upon the binding of envelope protein gp120, CD4 receptor physically associates with either CXCR4 or CCR5 receptors to initiate the formation of the HIV entry complex [11], [12]. CXCR4 or CCR5 can also form heterodimers with other GPCR receptors for initiation or alteration of signaling by these involved receptors. For example, CXCR4 and the δ-opioid receptor (DOR), both of which are expressed on the surface of monocytes and other immune cells, form heterodimers the presence of ligands for each receptor. The formation of the CXCR4:DOR heterodimer prevents each of them from signaling [6]. CCR5 and CCR2 can form heterodimers on the surface of the cells when they are stimulated with both CCL5 and CCL2 (ligands of CCR5 and CCR2, respectively) [5]. The CCR5:CCR2 heterodimers activate heterotrimeric G-protein Gq/11, instead of Gi, which is activated by CCR5 or CCR2 alone [5]. It appears that heterodimerization in response to chemokine binding is required for the termination or alteration of signaling by an increasing number of chemokine receptors [13].

CXCR4 and CCR5 are expressed on the surface of T lymphocytes and, during T cell activation, are both recruited to the immunological synapse (IS). This recruitment requires chemokine secretion by antigen-presenting cells (APCs) [14]. Therefore, it has been proposed that APC-derived chemokines promote formation of CXCR4:CCR5 heterodimers, resulting in accumulation of these receptors at the IS. Despite the important roles of CXCR4 and CCR5 in chemotaxis, HIV entry, and T cell activation, it is still not clear whether CXCR4 and CCR5 form heterodimers on the surface of live cells.

In this report, we investigated the formation of heterodimers between CXCR4 and CCR5 on the surface of live cells using FRET imaging coupled with quantitative microscopic analyses. CXCR4 was tagged with CFP (FRET donor), and CCR5 and two CCR5 mutants with altered C-termini, CCR5^STA^ and CCR5^Δ4^, were fused with YFP (FRET acceptor). We observed that CXCR4CFP, CCR5YFP, CCR5^STA^YFP and CCR5^Δ4^YFP could be expressed on the surface of live cells. When co-expressed, CXCR4CFP and CCR5YFP displayed a high level of FRET signal, indicating that CXCR4 and CCR5 formed heterodimers. In contrast, CXCR4CFP and CCR5^STA^YFP showed a low level of FRET signal and CXCR4CFP and CCR5^Δ4^YFP showed little FRET signal, suggesting that mutations in the C-terminus of CCR5 caused a decrease in CCR5's ability to form dimers with CXCR4. Furthermore, CCR5 chemokines, CCL3/MIP1α or CCL5/RANTES, induced a clear increase, while CXCR4 ligand, CXCL12/SDF-1, triggered a decrease in FRET between CXCR4CFP and CCR5YFP, suggesting that the binding of these chemokines differentially modulates the stability or conformation of CXCR4:CCR5 heterodimers in the plasma membrane.

## Materials and Methods

### Chemicals and reagents

pEYFP-N1 and pECFP-N1 were purchased from Clontech (Palo Alto, CA). Lipofectamine 2000 was purchased from Invitrogen (Grand Island, NY). CCL3/MIP1α, CCL5/RANTES and CXCL12/SDF1α were purchased from BioSource (Camarillo, CA). Fluo-4-AM was from Molecular Probes (Eugene, OR). Anti-GFP monoclonal antibody (JL-8) was from BD Biosciences Clontech (Palo Alto, CA). All of the other reagents were of reagent grade and were obtained from standard suppliers.

### Plasmid, cell line, and transfection

Human CXCR4 and CCR5 gene were generated by PCR. Plasmids carrying mutant receptors CCR5^STA^ and CCR5^Δ4^ were generous gifts from Dr. Murphy's group at NIAID, NIH. The plasmids encoding CCR5YFP, CCR5^STA^YFP and CCR5^Δ4^YFP were constructed by inserting the PCR product of CCR5, CCR5^STA^ and CCR5^Δ4^ into the pEYFP-N1 vectors in multicloning sites. The plasmid encoding CXCR4CFP was constructed by inserting the PCR product of CXCR4 into the pECFP-N1 vector. HEK293T cells were cultured in Dulbecco modified Eagle medium supplemented with fetal calf serum (10%), penicillin (5 µg/ml), and streptomycin (5 µg/ml) and were grown in 5% CO_2_ at 37°C. HEK293T cells were transfected or co-transfected with the plasmids encoding CCR5YFP, CCR5^STA^YFP, CCR5^Δ4^YFP and/or CXCR4CFP mediated by Lipofectamine 2000 (Invitrogen) according to the manufacturer's instructions [16].

### Calcium assay

HEK293T cells were seeded in four-well chambers at 10^4^/ml, 24–36 hrs before the experiments. After 3 hours of starvation, the cells were labeled by incubation with Fluo-4-AM in Hanks balanced salt solution for half an hour, washed twice, and incubated for half an hour before being taken images under the microscope. Upon the addition of SDF-1 (50 nM) or RANTES (50 nM) to the cell chamber, time-lapse images were collected with multi-track mode (Zeiss 510), and CFP and Fluo-4 images were digitally separated. Changes in Ca^2+^ concentration were represented as the changes in the intensity of Fluo-4 (I_t_/I_0_, where I_t_ is the intensity at time t, and I_0_ is the intensity at time 0) as previous described [7].

### Imaging and FRET assay

Cells were washed twice with 1×HBSS and then starved in 1×HBSS+1%BSA for 3 hrs. Before imaging, the cells were treated with chemokines for 20 min. Zeiss Plan-apochromat 40× oil immersion objective was used for image acquisition. Images were collected with multi-track mode (Zeiss 510). In Track I, there were two channels, cells were excited with 458 nm, CFP emission signals were collected through Channel I (475–525 nm) and FRET emission signals were collected through Channel II (>530 nm). In Track II, there was only one YFP channel, YFP emission signals were collected with this channel (>530 nm). FRET efficiency between CFP and YFP was analyzed using Zeiss LSM Software.

Intermolecular FRET efficiency was shown as N-FRET using macro of Zeiss LSM Software. Briefly, Sensitized Emission bleed-through (or crosstalk) coefficients were determined using control cells that expressed only CFP or YFP and expressed as correction factors as follows. Donor coefficients Fd/Dd: the amount of crosstalk of donor signal into the FRET channel. Ad/Fd: the amount of crosstalk of FRET signal into the Acceptor channel. Acceptor coefficients: Fa/Aa: the amount of crosstalk of the acceptor signal into the FRET channel. Da/Fa: the amount of crosstalk of the acceptor signal into the donor channel. Da/Fa: the amount of crosstalk of the FRET signal into the donor channel. Display N-FRET image with intensities that is converted from the FRET index is calculated for each pixel by LSM FRET tool for Carl Zeiss AIM software using the method Xia and Liu [17]. For quantification of N-FRET, regions of interest (ROIs) covering the plasma membrane from the acquired images were chosen, processed as above, and calculated automatically using the FRET macro of LSM imaging software as previously described [16]. Means and SD are shown. Statistical significance was determined with Student's t-test.

## Results

### Expression of CXCR4, CCR5, and mutant CCR5 receptors tagged with fluorescent proteins

**Figure 1:**
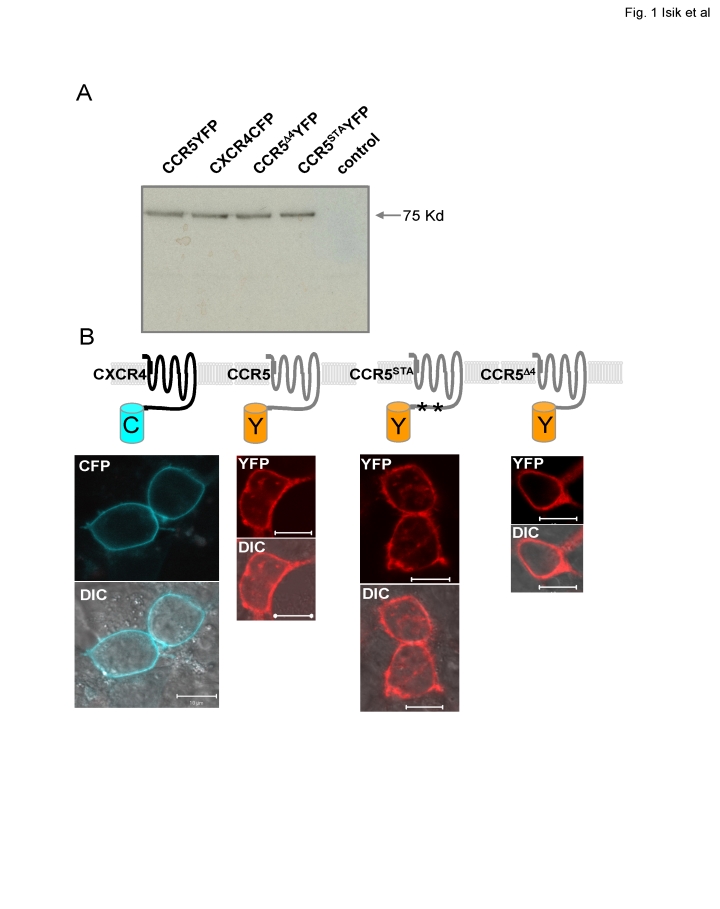
Expression of CXCR4 or CCR5 and CCR5 mutant receptors tagged with fluorescent proteins. A. The indicated CFP- or YFP-tagged receptors, transiently expressed in HEK293 cells, were detected in whole cell lysates by western blotting with an anti-GFP antibody. Untransfected HEK293 cells were included as a negative control. B. Schematic diagram of CXCR4CFP, CCR5YFP, CCR5^STA^YFP and CCR4^Δ4^YFP on the cell membrane. Confocal images of living cells expressing membrane-localized CXCR4CFP (cyan), CCR5YFP (red), CCR5^STA^YFP (red) and CCR5^Δ4^YFP (red); top panels, fluorescence images. Bottom panels, the merged images of the fluorescence and differential-interference-contrast (DIC) images. Scale bar, 10 µm.

To investigate the distributions of CXCR4 and CCR5 in the plasma membrane of live cells, we fused CFP to the C-terminus of CXCR4, and YFP to the C-terminus of CCR5. It is well known that upon activation of CCR5 the C-terminal tail interacts with GPCR kinase(s) and arrestin to carry out receptor functions [9]. In this study, we selected two CCR5 mutants, CCR5^STA^ and CCR5^Δ4^, and fused YFP to their C-termini. CCR5^STA^ is a CCR5 mutant in which all serines and threonines in the C-terminal tail were replaced by alanines; while CCR5^Δ4^ is a mutant in which the last 46 amino acids (307–352 a.a.) of the C-terminus were removed [15]. HEK293 cells were transfected with the CXCR4CFP, CCR5YFP, CCR5^Δ4^YFP or CCR5^STA^YFP constructs and expression of appropriately sized fusion proteins was verified by western blotting with anti-GFP antibodies (Figure 1A). Fluorescence microscopy revealed that the majority of CXCR4CFP, CCR5YFP, CCR5^STA^YFP was expressed uniformly on the plasma membrane (Figure. 1B). The surface expression of CCR5^Δ4^YFP, on the other hand, was less efficient compared to the other tagged receptors. Consistent with a previous report [15], CCR5^Δ4^YFP was frequently localized to the interior of transfected cells (data not shown). However, it was expressed on the cell surface in a small fraction of the cells under our experimental condition, which allowed us to carry out the measurement of CCR5^Δ4^YFP on the surface of live cells (Figure 1B).

### Functional characterization of the tagged receptors

**Figure 2:**
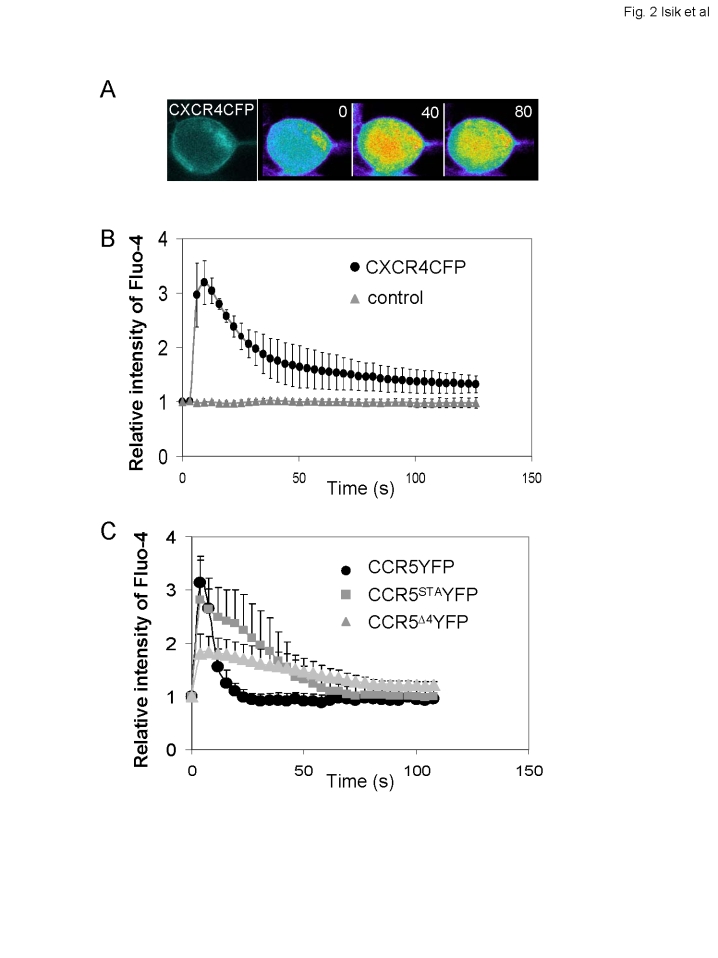
Ligand-induced Ca^2+^ response in living cells expressing CXCR4CFP, CCR5YFP, CCR5^STA^YFP or CCR5^Δ4^YFP. A. CXCR4CFP mediated a transient intracellular Ca^2+^ elevation, which is visualized as intensity changes of Fluo-4 (rainbow color). SDF-1 was added at time 0. B. A time course of intracellular Ca^2+^ changes following the addition of SDF-1 at time 0. SDF-1-induced Ca^2+^ response in cells expressing CXCR4CFP (black circles) (n = 7) or HEK293 cells as a control (gray triangles) (n = 4). Means±S.D. are shown. C. A time course of intracellular Ca^2+^ changes following the addition of MIP1α, a ligand of CCR5, at time 0. MIP1α-induced Ca^2+^ responses in cells expressing CCR5 (black circles) (n = 4), CCR5^STA^YFP (gray squares) (n = 6) and CCR5^Δ4^YFP (gray triangles) (n = 10). Means±S.D. are shown.

To test the functionality of CXCR4CFP, we examined ligand-induced Ca^2+^ responses in the cells that expressed CXCR4CFP on the cell surface (Figure 2A). We imaged fluorescence intensity change of Fluo-4, a calcium indicator, triggered by the CXCR4 ligand SDF-1. Using a confocal fluorescence microscope (Zeiss 510 META), fluorescence images of CXCR4CFP and Fluo-4 were simultaneously recorded in a time-lapse experiment (Figure 2A). CXCR4CFP primarily localized to the cell surface, while Fluo-4 was distributed throughout the cytosol. Upon addition of SDF-1 to the cell chamber, the Fluo-4 fluorescence signal transiently increased in the cytosol of the CXCR4CFP cells but not that of the parental HEK293 cells, indicating that the CXCL12/SDF-1-elicited Ca^2+^ response was specifically mediated by the expressed CXCR4CFP (Figure 2A and 2B). Our previous study demonstrated that the CCR5 receptor fused with CFP at its C-terminus is also functional in the ligand-induced Ca^2+^ response [16]. We also investigated ligand-induced Ca^2+^ response in cells expressing CCR5^STA^YFP and CCR5^Δ4^YFP under the same live cell imaging conditions (Figure 2C). Upon addition of MIP1α, a ligand for CCR5, to the cell chamber, Fluo-4 fluorescence signal increased in the cells expressing CCR5-YFP, CCR5^STA^YFP and CCR5^Δ4^YFP, indicating that these tagged receptors also retained their ligand-binding and signaling functions (Figure 2C). However, CCR5^STA^YFP and CCR5^Δ4^YFP triggered the Ca^2+^ responses with different kinetics compared with that induced by CCR5YFP, indicating that mutations of the C-terminal tail of CCR5 affected its ability in signaling (Figure 2C). Taken together, cells expressing CXCR4CFP, CCR5YFP, CCR5^STA^YFP and CCR5^Δ4^YFP provide a system for probing extracellular ligand-induced changes in the dynamics distribution of these receptors on plasma membrane of live cells.

### Co-localization of CXCR4 and CCR5 or CCR5 mutant receptors

**Figure 3:**
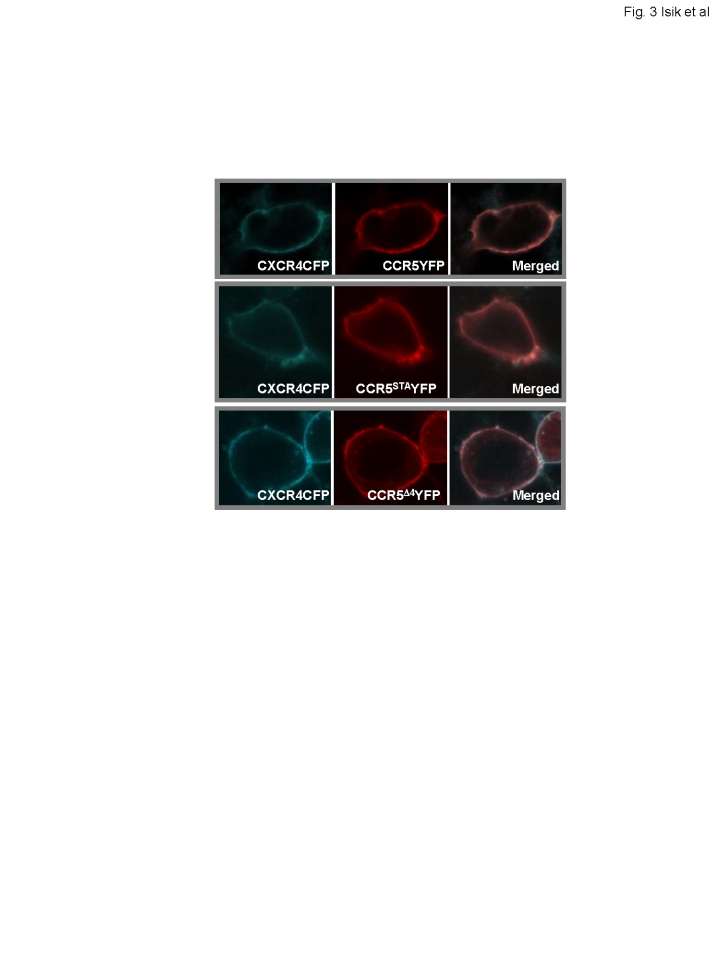
Co-localization of CXCR4 and CCR5 or CCR5 mutants on the surface of living cells. HEK293 cells were co-transfected with CFP-tagged CXCR4 and YFP-tagged CCR5, or CCR5 mutants. Fluorescence images show distribution of CXCR4CFP (cyan) and CCR5YFP, CCR5STAYFP or CCR5Δ4YFP (red). The merged images are presented to show co-localization.

We investigated membrane distribution of CXCR4 and CCR5 or CCR5 mutants in HEK293 cells that co-expressed CXCR4CFP and either CCR5YFP, CCR5^STA^YFP or CCR5^Δ4^YFP. Using multitrack and line-scanning mode of a laser-scanning confocal microscope, cells were simultaneously recorded in the CFP and YFP detection channel. We observed that CXCR4CFP co-localized on the cell surface with each of the CCR5 variants (Figure 3). However, this co-localization does not prove that the receptors are physically associated given that the spatial resolution of light microscopy is more than 200 nm. Therefore, we used the FRET imaging method to determine if CXCR4CFP was in close proximity to CCR5YFP, CCR5^STA^YFP and CCR5^Δ4^YFP on the cell surface.

### CXCR4 and CCR5 exist as preformed dimers on the cell surface

**Figure 4:**
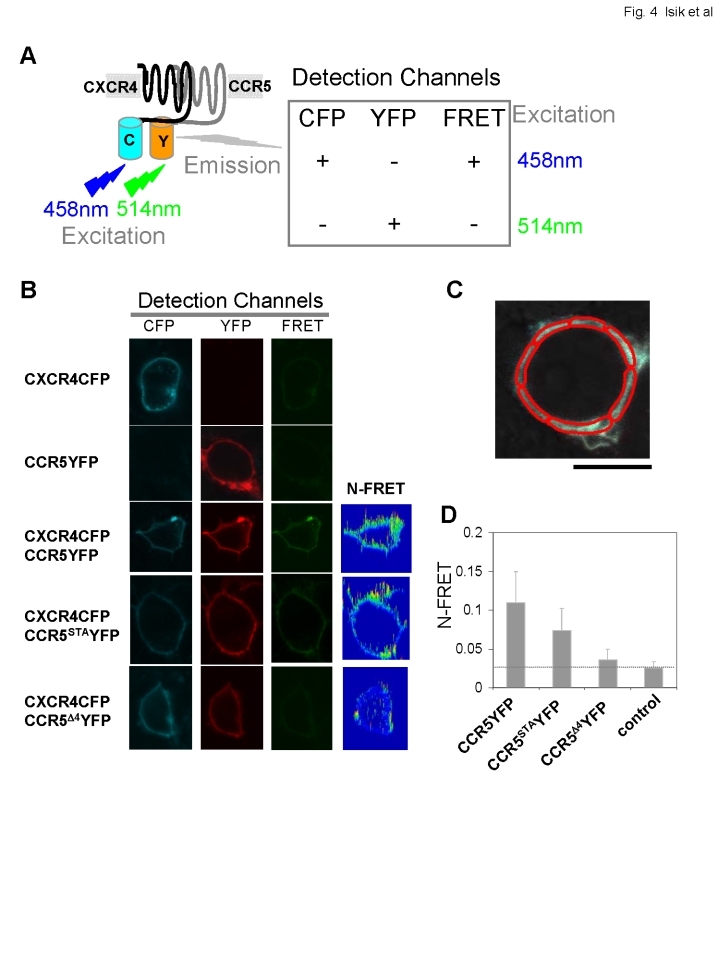
An analysis of CXCR4 and CCR5 association on the surface of live cells by FRET imaging. A. Schematic diagram of FRET measurement between CFP-tagged CXCR4 and YFP-tagged CCR5. When cells were excited at 458 nm, emissions are simultaneously recorded in CFP channel and FRET channel, and when cells were excited at 514 nm, emissions were recorded in YFP channels. We used multi-channel and line-scanning mode so that images of three channels were recorded simultaneously. Cells expressing only CXCR4CFP or CCR5YFP were used as controls for calculating real FRET efficiency that is expressed as normalized FRET (N-FRET). B. Images of cells expressing only CXCR4CFP or CCR5YFP; CXCR4CFP with CCR5YFP, and CCR5^STA^YFP or CCR5^Δ4^YFP. N-FRET images show FRET intensities. C. FRET efficiency, N-FRET, in the plasma membrane was measured in selected regions of interest (ROIs). One example is shown. D. Quantitative analysis of N-FRET on cells expressing CXCR4CFP and CCR5YFP (n = 198), CCR5^STA^YFP (n = 156) or CCR5^Δ4^YFP (n = 148) is shown as means±S.D. Cells expressing IL17RA-YFP and TNFR-CFP (n = 58) were used as negative control. Statistical significance was assessed using a t-test.

To examine interactions between CXCR4 and CCR5, we measured FRET between CFP and YFP in cells co-expressing CXCR4CFP and CCR5YFP. For this we used confocal microscopy and the sensitized emission method to calculate FRET efficiency between the CFP and YFP moieties. Using a multitrack and line-scanning mode of a laser-scanning confocal microscope, cells were simultaneously recorded in the CFP and YFP, and FRET detection channels. Fluorescence was simultaneously collected pixel-by-pixel from three detection channels: CFP (458 nm, CFP excitation, 475–525 nm, CFP emission); FRET (458 nm, CFP excitation; long pass filter 530 nm, YFP emission); and YFP (514 nm, YFP excitation, long pass filter 530 nm, YFP emission) (Figure 4A). We first obtained bleed-through (cross talk) co-efficient by analyzing images from cells expressing only CXCR4CFP or CCR5YFP, which were imaged with the identical configuration and scanning setup as the controls (Figure 4B). We then obtained normalized FRET (N-FRET) efficiency in the plasma membrane of cells expressing both CXCR4CFP and CCR5YFP. Using the FRET analysis tool, the FRET macro for the Ziess LSM 510 META microscope, the normalized FRET (N-FRET) image with intensities was converted from the FRET index calculated from each pixel as previously described [16], [17] (details in Materials and Methods).

An advantage of using confocal microscopy to evaluate FRET is that individual region of interests (ROIs) within a cell can be selectively examined for FRET efficiency [18]. In contrast, fluorometric [19] or flow cytometric [20], [21] approaches can only measure total cellular FRET, which often includes concentrations of fluorophores in intracellular compartments. Because we were interested in CXCR4 and CCR5 interactions at the cell surface, only the plasma membrane region of the cell was selected as the ROIs (Figure 3C). FRET efficiency is usually sensitive to the relative amounts of donors and acceptors in the selected regions. However, we took the following steps to minimize the possibility that our measurements were strongly influenced by high levels of the receptor expression. First, all the FRET images were recorded using identical parameters, including excitation laser intensity, detector gains and magnification. Therefore, the levels of CXCR4CFP and CCR5YFP, CCR5^STA^YFP or CCR5^Δ4^YFP were clearly monitored pixel-by-pixel by CFP and YFP intensities. Second, in our analyses, we selected regions having mean CFP and YFP intensities between 600 and 2800 to ensure the proper receptor levels. Finally, FRET values were normalized pixel-by-pixel by dividing by the square root of the donor and acceptor concentrations to yield N-FRET values. We have previously shown that such N-FRET values are relatively independent of the CFP or YFP concentrations and, therefore, likely reflect receptor interactions [16], [22].

We used tagged receptors for IL17 and TNF (IL17RA-YFP and TNFR-CFP), which both localize to the cell membrane and do not associate as a receptor complex, as negative controls for determining the baseline for FRET efficiency [20], [22]. Strikingly, cells expressing CXCR4CFP and CCR5YFP showed a marked enhancement of FRET (Figure 4B and 4D). In clear contrast, cells that expressed membrane-localized CXCR4CFP and CCR5^Δ4^YFP showed very low FRET intensity, while cells expressing CXCR4CFP and CCR5^STA^YFP displayed moderate FRET signal (Figure 4B and 4D). Quantitative analyses of FRET efficiency indicated that normalized FRET (N-FRET) between CXCR4CFP:CCR5YFP (n = 198) was significantly greater than in the case of CXCR4CFP:CCR5^Δ4^YFP (n = 148, p<0.01) or the negative control (n = 58, p<0.01) (Figure 4D). In addition, the intermediate N-FRET value obtained for CXCR4CFP:CCR5^STA^YFP (n = 156) was significantly lower than that of CXCR4CFP:CCR5YFP (p<0.01) but still higher than those of CXCR4CFP:CCR5^Δ4^YFP (p<0.01) and the negative control (p<0.01). On the other hand, N-FRET of CXCR4CFP:CCR5^Δ4^YFP did not significantly differ from that of the control (p>0.01). Together, these data suggest that CCR5 dimerizes with CXCR4 on the plasma membrane in the absence of ligands and, moreover, that the C-terminal tail mutations of CCR5^STA^ or CCR5^Δ4^ reduce or abolish the receptor's ability to form pre-existing dimers with CXCR4.

### Ligand binding to CCR5 or CXCR4 differentially affects heterodimers

**Figure 5:**
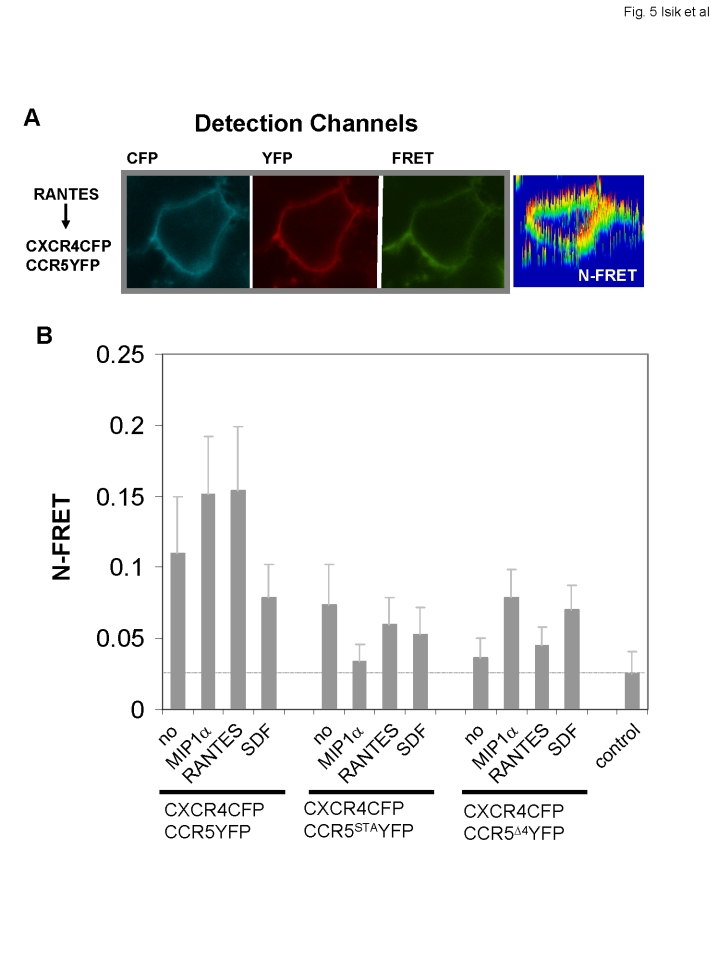
Ligand-binding induced FRET changes between heterodimers. A. A representative image of a cell expressing CXCR4CFP and CCR5YFP that was stimulated with RANTES. The N-FRET image shows marked FRET signals. B. Cells expressing CXCR4CFP and CCR5YFP, or CCR5^STA^YFP or CCR5^Δ4^YFP were stimulated with ligands such as CCL3/MIP1α, CCL5/RANTES, or CXCL12/SDF-1. Quantitative analyses of N-FRET under each condition. Data for N-FRET are shown as means±S.D. Statistically significant N-FRET changes are indicated as p-values in the results.

To examine the effects of CCR5 and CXCR4 ligands on the pre-existing dimers, we measured the FRET efficiency between CXCR4CFP and CCR5YFP upon stimulation with CCL3/MIP1α and CCL5/RANTES (ligands of CCR5) and CXCL12/SDF-1 (a ligand of CXCR4). Relative to the unstimulated control, both of the CCR5 ligands induced N-FRET increases between CXCR4CFP and CCR5YFP of roughly 30% (p<0.01 for both), while CXCL12/SDF-1 (n = 119) triggered a slight decrease in the N-FRET signal (p<0.01) (Figure 5A and 5B) These results suggest that ligands binding to each receptor comprising the heterodimer distinctly alter its conformation. In contrast to the results obtained with CCR5YFP, addition of CCL3/MIP1α (n = 105) resulted in a decrease in N-FRET (p<0.01) while CCL5/RANTES (n = 97) did not cause a significant change (p>0.05) in N-FRET between CXCR4CFP and CCR5^STA^YFP (n = 156 with no stimulation) (Figure 5). Interestingly, while CCR5^Δ4^YFP could hardly form dimers with CXCR4CFP (n = 148) in the absence of ligands, addition of CCL3/MIP1α (n = 85) and CXCL12/SDF-1 (n = 96) caused clear increases in N-FRET between these receptors (p<0.01 for both), suggesting that binding of either CXCL12/SDF-1 to CXCR4 or CCL3/MIP1α (but not CCL5/RANTES n = 138, p>0.05) to CCR5^Δ4^ promotes the formation of CXCR4:CCR5^Δ4^ heterodimers. Taken together, our results suggest that the formation of heterodimers between CXCR4 and CCR5 is a dynamic process and that ligand binding may cause conformational changes in pre-existing CXCR4:CCR5 dimers, destabilize CXCR4:CCR5^STA^ heterodimers or promote CXCR4:CCR5^Δ4^ heterodimer formation.

## Discussion

In recent years, a number of GPCRs have been shown to exist as dimers in the plasma membrane, raising a number of interesting questions regarding the molecular dynamics and functional significance of receptor dimer formation [9]. One of the highly debated questions is whether dimerization in the cell membrane occurs spontaneously or is induced by ligand binding. The chemokine receptors CXCR4 and CCR5 regulate leukocyte chemotaxis and are also instrumental in the entry of HIV into immune cells [12], [23]. However, their dimerization properties have not been clearly defined. In this study using a fluorescence resonance energy transfer imaging, we provide the first evidence that CXCR4 and CCR5 form heterodimers on the plasma membrane in the absence of chemokines and that their chemokine ligands induced different conformational changes that either promote or destabilize the formation of heterodimers.

Our results indicate that CXCR4 and CCR5 heterodimers exist on the surface of cells in the absense of ligand stimulation and that point mutations within or deletion of the C-terminus of CCR5 reduce the ability of the receptor to form heterodimers with CXCR4 (Fig. 3). CCR5 has been previously shown to form homodimers and heterodimers with CCR2 chemokine receptor [5]. Previous studies indicated that transmembrane regions TM1, TM2 and TM4 of CCR5 are involved in the homodimer formation. Several amino acids, such as Ile52 in TM1 and Val150 in TM4, have been shown to be crucial for CCR5 function and homodimerization [24]. The role of the C-terminus of CCR5 has been examined in receptor surface expression, receptor signaling and HIV entry. The C-terminal deletion mutants reduced the surface expression [15], [25], [26]. We found the CCR5^STA^ mutant was expressed on the cell surface, and its chemokine-induced Ca^2+^ response prolonged, which is consistent with the defect of the receptor in desensitization that is required for phosphorylation at C-terminus [15]. We also found that the deletion mutant CCR5^Δ4^ was aberrantly expressed in the cytosol in a majority of transfected cells, but surface expression of CCR5^Δ4^ was detected in a small number of cells. Using live single cell imaging, we were able to use these mutants to analyze functions of the C-terminus in chemokine-induced Ca^2+^ responses and heterodimer formation. Our data suggested that the C-terminal tail of CCR5 may be involved in the formation of CXCR4 and CCR5 heterodimers.

Our data revealed that ligand binding to CCR5 or CXCR4 could either promote or destabilize the formation of heterodimers. Previous studies indicated that ligand binding could promote the formation of homo- or heterodimers. For example, the CXCR4 and δ-opioid receptor (DOR) form both homo- and heterodimers and extracellular ligands could alter the complexes of these dimers [6]. DPDPE, a ligand of DOR, induces DOR homodimers; CXCL12/SDF-1, a ligand of CXCR4, triggers the formation of CXCR4 homodimers; and DPDPE and SDF-1 together promote assembly of heterodimeric CXCR4:DOR complexes. It has been proposed that the formation of homo- or heterodimers on the cell membrane, where various receptors co-exist, is a dynamic process, and various ligands bind to their respective receptors and alter the complexes of receptors. Our study indicated a new level of complexity regarding to the effect of ligand binding on the formation of receptor heterodimers. While binding of CCL3/MIP1α and CCL5/RANTES to CCR5 promoted the formation of CCR5:CXCR4 heterodimers, CXCL12/SDF-1 binding to CXCR4 reduced the level of preexisting CCR5:CXCR4 heterodimers. Mutations in the C-terminal tail of CCR5 not only reduced or abolished its ability to form heterodimers with CXCR4 but also altered effects of ligand binding on the formation of heterodimers with CXCR4. CCL3/MIP1α binding to CCR5^STA^ mutant receptor reduced its ability to form heterodimer with CXCR4, while the binding of CCL5/RANTES to CCR5 or CXCL12/SDF-1 to CXCR4 had no apparent effect. Furthermore, CCL3/MIP1α and CXCL12/SDF-1 binding to either CCR5^Δ4^ mutant or CXCR4 promoted formation of heterodimers. Our results suggest that ligand binding to a chemokine receptor causes a conformational change in the receptor that can either promote or destabilize the formation of receptor dimers. Mutations of the C-terminal domain of a receptor affect ligand-induced conformational changes, thereby altering its potential to form dimers.

Our results have clear implication for the in vivo physiology of chemokine and their receptors. It has been suggested that chemokines secreted by antigen-presenting cells recruit both CXCR4 and CCR5 to the immunological synapse (IS) during T cell activation [14]. Our data suggest that this accumulation of both CXCR4 and CCR5 could be mediated by chemokine-promoted formation of CXCR4:CCR5 heterodimers. Our results also provide information on the functions of the C-terminal tail of chemokine receptors in dimerization and thus define additional targets for potential drugs in chemokine-related diseases. It should be noted that our study was performed using HEK293 cells expressing CFP- or YFP-tagged receptors. Whether or not the CXCR4:CCR5 dimerization and ligand-induced changes we observed occur in actual leukocytes in vivo remains to be determined. New techniques are to be developed in order to apply this imaging approach in vivo [27].

## References

[1] Murphy PM (1994). The molecular biology of leukocyte chemoattractant receptors.. Annu Rev Immunol.

[2] Murphy PM, Baggiolini M, Charo IF, Hebert CA, Horuk R (2000). International union of pharmacology. XXII. Nomenclature for chemokine receptors.. Pharmacol Rev.

[3] Moser B, Loetscher P (2001). Lymphocyte traffic control by chemokines.. Nat Immunol.

[4] Soriano SF, Serrano A, Hernanz-Falcon P, Martin de Ana A, Monterrubio M (2003). Chemokines integrate JAK/STAT and G-protein pathways during chemotaxis and calcium flux responses.. Eur J Immunol.

[5] Mellado M, Rodriguez-Frade JM, Vila-Coro AJ, Fernandez S, Martin de Ana A (2001). Chemokine receptor homo- or heterodimerization activates distinct signaling pathways.. EMBO J.

[6] Pello OM, Martinez-Munoz L, Parrillas V, Serrano A, Rodriguez-Frade JM (2008). Ligand stabilization of CXCR4/delta-opioid receptor heterodimers reveals a mechanism for immune response regulation.. Eur J Immunol.

[7] Jiao X, Zhang N, Xu X, Oppenheim JJ, Jin T (2005). Ligand-induced partitioning of human CXCR1 chemokine receptors with lipid raft microenvironments facilitates G-protein-dependent signaling.. Mol Cell Biol.

[8] Strange PG (2008). Signaling mechanisms of GPCR ligands.. Curr Opin Drug Discov Devel.

[9] Gurevich VV, Gurevich EV (2008). GPCR monomers and oligomers: it takes all kinds.. Trends Neurosci.

[10] Lazzarino DA, Diego M, Musi E, Hirschman SZ, Alexander RJ (2000). CXCR4 and CCR5 expression by H9 T-cells is downregulated by a peptide-nucleic acid immunomodulator.. Immunol Lett.

[11] Berger EA, Murphy PM, Farber JM (1999). Chemokine receptors as HIV-1 coreceptors: roles in viral entry, tropism, and disease.. Annu Rev Immunol.

[12] Murphy PM (2001). Viral exploitation and subversion of the immune system through chemokine mimicry.. Nat Immunol.

[13] Hereld D, Jin T (2008). Slamming the DOR on chemokine receptor signaling: heterodimerization silences ligand-occupied CXCR4 and delta-opioid receptors.. Eur J Immunol.

[14] Molon B, Gri G, Bettella M, Gomez-Mouton C, Lanzavecchia A (2005). T cell costimulation by chemokine receptors.. Nat Immunol.

[15] Venkatesan S, Petrovic A, Locati M, Kim YO, Weissman D (2001). A membrane-proximal basic domain and cysteine cluster in the C-terminal tail of CCR5 constitute a bipartite motif critical for cell surface expression.. J Biol Chem.

[16] Yi L, Fang J, Isik N, Chim J, Jin T (2006). HIV gp120-induced interaction between CD4 and CCR5 requires cholesterol-rich microenvironments revealed by live cell fluorescence resonance energy transfer imaging.. J Biol Chem.

[17] Xia Z, Liu Y (2001). Reliable and global measurement of fluorescence resonance energy transfer using fluorescence microscopes.. Biophys J.

[18] Xu X, Meier-Schellersheim M, Yan J, Jin T (2007). Locally controlled inhibitory mechanisms are involved in eukaryotic GPCR-mediated chemosensing.. J Cell Biol.

[19] Janetopoulos C, Jin T, Devreotes P (2001). Receptor-mediated activation of heterotrimeric G-proteins in living cells.. Science.

[20] Siegel RM, Frederiksen JK, Zacharias DA, Chan FK, Johnson M (2000). Fas preassociation required for apoptosis signaling and dominant inhibition by pathogenic mutations.. Science.

[21] Siegel RM, Chan FK, Zacharias DA, Swofford R, Holmes KL (2000). Measurement of molecular interactions in living cells by fluorescence resonance energy transfer between variants of the green fluorescent protein.. Sci STKE.

[22] Kramer JM, Yi L, Shen F, Maitra A, Jiao X (2006). Evidence for ligand-independent multimerization of the IL-17 receptor.. J Immunol.

[23] Mellado M, Rodriguez-Frade JM, Manes S, Martinez AC (2001). Chemokine signaling and functional responses: the role of receptor dimerization and TK pathway activation.. Annu Rev Immunol.

[24] Hernanz-Falcon P, Rodriguez-Frade JM, Serrano A, Juan D, del Sol A (2004). Identification of amino acid residues crucial for chemokine receptor dimerization.. Nat Immunol.

[25] Doranz BJ, Lu ZH, Rucker J, Zhang TY, Sharron M (1997). Two distinct CCR5 domains can mediate coreceptor usage by human immunodeficiency virus type 1.. J Virol.

[26] Gosling J, Monteclaro FS, Atchison RE, Arai H, Tsou CL (1997). Molecular uncoupling of C-C chemokine receptor 5-induced chemotaxis and signal transduction from HIV-1 coreceptor activity.. Proc Natl Acad Sci USA.

[27] Jin T, Xu X, Hereld D (2008). Chemotaxis, chemokine receptors and human disease.. Cytokine.

